# Long-Term Overexpression of Hsp70 Does Not Protect against Cardiac Dysfunction and Adverse Remodeling in a MURC Transgenic Mouse Model with Chronic Heart Failure and Atrial Fibrillation

**DOI:** 10.1371/journal.pone.0145173

**Published:** 2015-12-14

**Authors:** Bianca C. Bernardo, Geeta Sapra, Natalie L. Patterson, Nelly Cemerlang, Helen Kiriazis, Tomomi Ueyama, Mark A. Febbraio, Julie R. McMullen

**Affiliations:** 1 Baker IDI Heart and Diabetes Institute, Melbourne, 3004, Australia; 2 Department of Cardiovascular Medicine, Graduate School of Medical Science, Kyoto Prefectural University of Medicine, Kyoto 602–8566, Japan; 3 Garvan Institute of Medical Research, Darlinghurst, 2010, Australia; University of Cincinnati, College of Medicine, UNITED STATES

## Abstract

Previous animal studies had shown that increasing heat shock protein 70 (Hsp70) using a transgenic, gene therapy or pharmacological approach provided cardiac protection in models of acute cardiac stress. Furthermore, clinical studies had reported associations between Hsp70 levels and protection against atrial fibrillation (AF). AF is the most common cardiac arrhythmia presenting in cardiology clinics and is associated with increased rates of heart failure and stroke. Improved therapies for AF and heart failure are urgently required. Despite promising observations in animal studies which targeted Hsp70, we recently reported that increasing Hsp70 was unable to attenuate cardiac dysfunction and pathology in a mouse model which develops heart failure and intermittent AF. Given our somewhat unexpected finding and the extensive literature suggesting Hsp70 provides cardiac protection, it was considered important to assess whether Hsp70 could provide protection in another mouse model of heart failure and AF. The aim of the current study was to determine whether increasing Hsp70 could attenuate adverse cardiac remodeling, cardiac dysfunction and episodes of arrhythmia in a mouse model of heart failure and AF due to overexpression of Muscle-Restricted Coiled-Coil (MURC). Cardiac function and pathology were assessed in mice at approximately 12 months of age. We report here, that chronic overexpression of Hsp70 was unable to provide protection against cardiac dysfunction, conduction abnormalities, fibrosis or characteristic molecular markers of the failing heart. In summary, elevated Hsp70 may provide protection in acute cardiac stress settings, but appears insufficient to protect the heart under chronic cardiac disease conditions.

## Introduction

Heat shock proteins (Hsps) are endogenous proteins that are upregulated by a wide range of stress conditions, and have been considered potential therapeutic targets in cardiac disease [1–3]. The best recognized function of Hsps is acting as intracellular chaperones for other proteins, playing an essential role for correct protein function (via folding and correct protein conformational shape), and preventing unwanted protein aggregation which occurs with aging and in settings of cardiac stress [4, 5]. The most conserved and best studied class of all heat shock proteins is the 70kDa family [2]. This study focuses on the most abundant and well characterized stress-inducible isoform of the 70kDa family which we have referred to as Hsp70 (also known as Hsp72). A number of earlier animal studies had suggested that increasing Hsp70 in the heart provides protection in settings of ischemia and atrial fibrillation (AF). Isolated hearts from transgenic (Tg) mice overexpressing Hsp70 were protected in various models of ischemia [6–10], and gene delivery of Hsp70 also improved outcome in rat and rabbit hearts subjected to ischemia-reperfusion [11–13]. Geranylgeranylacetone (GGA), a Hsp inducer which increases Hsp70, was shown to prevent ischaemia-induced atrial conduction abnormalities and suppressed ischaemia-related AF in dogs [14]. Clinical based research had also provided evidence to link Hsp70 expression in human cardiac tissue with protection against AF. For instance, patients with high Hsp70 expression levels displayed a lower incidence of postoperative AF, whereas an M439T amino acid substitution in Hsp70 was associated with an increased risk of postoperative AF [15–17]. AF is the most common rhythm disturbance in the heart and is associated with increased rates of heart failure (HF) and stroke. The prevalence of AF is increasing with rising rates of obesity, diabetes, and a growing aging population [18]. Given current treatments have limited efficacy, dangerous side effects, and poor compliance [19–21], investigations into new approaches are urgently required.

We recently investigated the effect of increasing Hsp70 expression using a transgenic approach in a mouse model that develops HF and displays intermittent AF [22]. Despite the previous promising animal studies and association studies in humans with Hsp70, we found no benefit of increasing Hsp70 expression in our HF model. The mechanism by which the mouse model developed HF and AF was due to transgenic overexpression of mammalian sterile 20-like kinase 1 (Mst1; a kinase activated by clinically important pathologic insults such as ischemia/reperfusion which leads to activation of caspases, apoptosis, and dilated cardiomyopathy [23]) together with a decrease in the cardioprotective kinase, phosphoinositide 3-kinase (PI3K; due to expression of a dominant negative PI3K (dnPI3K) mutant [24]). As previous reports had suggested that Hsp70 provides protection in the animal and human heart, we considered it important to assess the therapeutic potential of increasing Hsp70 in an additional mouse model which develops HF and AF due to a different mechanism of action.

The aim of the current study was to examine whether transgenic overexpression of Hsp70 could attenuate adverse cardiac remodeling in a mouse model which develops HF and AF due to the overexpression of Muscle-Restricted Coiled-Coil (MURC). The MURC transgenic mouse model was selected because it is one of the few available mouse models which develop AF spontaneously (i.e. display AF without the need for pacing). Furthermore, MURC was shown to activate the Rho/ROCK pathway which has also been implicated in inducing AF [25, 26]. RhoA and MURC transgenic mice have similar cardiac phenotypes [25, 26]; an interaction between MURC and SDPR, a phosphatidylserine-binding protein, has been implicated in activating RhoA [25]. RhoA signaling induces cardiac dysfunction and conduction disturbances by regulating cytoskeletal reorganization, gene expression, and cell death [25, 26]. Here, we report that the overexpression of Hsp70 was unable to attenuate pathological remodeling, improve cardiac function or reduce episodes of arrhythmia in the MURC Tg mouse model of HF and AF.

## Material and Methods

### Experimental animals

All animal experiments were carried out in accordance with the Australian code for the care and use of animals for scientific purposes (National Health and Medical Research Council of Australia, 8^th^ Edition, 2013). Animal care and experimental procedures were approved by the Alfred Medical Research and Education Precinct’s Animal Ethics Committee. Mice were housed in a temperature- controlled environment on a 12 hour light–dark cycle. Here, we used the cardiac specific MURC Tg mouse model as these mice develop HF and display spontaneous episodes of AF due to increased RhoA signaling; RhoA activity was elevated by approximately 2.1 fold in hearts of MURC Tg mice [25]. To examine whether Hsp70 may provide protection in the MURC HF model, MURC-Hsp70 Tg mice were generated. For this, female hemizygous Hsp70 Tg mice that overexpress Hsp70 in heart and skeletal muscle under the β-actin promoter and a cytomegalovirus enhancer (BALB/c background) [9] were bred with male MURC Tg mice (C57BL/6 background). Functional assessment and tissue collection was performed on male littermate non-transgenic (Ntg), MURC and MURC-Hsp70 Tg mice at approximately 12 months of age (all on the same genetic background: BALB/c-C57BL/6). A subset of gender-matched MURC Tg and Ntg mice were characterized at 16 weeks of age to examine whether these mice could be morphologically distinguished at this earlier time point. No gender differences were identified. An additional cohort of young (8–12 week) and old (12 months) Hsp70 Tg were characterized and compared to gender matched Ntg to investigate the impact of long-term chronic Hsp70 expression alone in the heart i.e. in the absence of MURC expression.

### Left ventricule (LV) structure and function

Twelve month old mice were anaesthetized (1.8% isoflurane, inhalation) and transthoracic echocardiography (M-mode two-dimensional echocardiography) was performed using a Philips iE33 ultrasound machine with a 15MHz linear array transducer. LV chamber dimensions (LV end-diastolic dimension, LVEDD; LV end-systolic dimension, LVESD), LV wall thicknesses (LV posterior wall, LVPW; interventricular septum, IVS), fractional shortening (FS, calculated as [(LVEDD–LVESD)/LVEDD] × 100%) and heart rate (HR) were evaluated from M-mode traces using the ProSolv Cardiovascular Analyzer version 3.5 software package (ProSolv, Indianapolis, IN).

### Electrocardiography (ECG)

Mice were anaesthetized (1.8% isoflurane) and ECGs were recorded by placing two 27-G needle electrodes subcutaneously (right arm and chest lead equivalent to V5). ECG readouts were collected for at least 5 minutes using the Powerlab system and BioAmp (ADInstruments). ECG parameters (PR interval-length of time from the beginning of the P-wave to the beginning of the QRS complex (representing atrioventricular conduction), RR interval-interbeat intervals, QRS interval-duration of the QRS complex, R-amplitudes and HR were analyzed using Chart 5 software (ECG analysis module). Arrhythmia was assessed (based on a change in HR>30 bpm) across the entire recording period, as we described previously [22].

### RNA and protein isolation

Total RNA was extracted from frozen mouse ventricles using TRI Reagent (Sigma-Aldrich, St Louis, MO) and quantitated with a Nanodrop™ Spectrometer (Thermo Scientific, Waltham, MA). To generate protein lysates, frozen mouse ventricles or atria were homogenized in lysis buffer (10% glycerol, 137 mM NaCl, 20 mM Tris-HCl (pH 7.4), 20 mM NaF, 10 mM EDTA, 1 mM EGTA, 1 mM sodium pyrophosphate, 1 mM vanadate, 1 mM PMSF, 4 μg/mL pepstatin, 4 μg/mL aprotinin, 4 μg/mL leupeptin) and the Bradford method (Biorad) was used to measure total protein concentration.

### Quantitative PCR (qPCR)

To assess mRNA expression, the High Capacity RNA-to-cDNA kit (Life Technologies, Carlsbad, CA) was used to reverse transcribe 2 μg of total RNA. qPCR was performed on 25ng/sample cDNA using TaqMan® probes (Life Technologies) and amplified with an Applied Biosystems Quant Studio 7 Flex real-time PCR instrument. Data was analyzed using the 2^-ΔΔCt^ quantification method. A list of TaqMan® probes are provided in S1 Table.

### Western blotting

Protein lysate samples were separated by SDS–polyacrylamide gel electrophoresis and blotted onto a polyvinylidene difluoride membrane (Merck). Membranes were incubated with primary antibody for at least 16 hours at 4°C, used at the following concentrations: 1:1000 Hsp70 (Stressgen, 810B), 1:5000 GAPDH (Santa Cruz, sc-32233), 1:1000 RhoA (Cell Signaling, #2117), 1:1000 Collagen 1 (Abcam, ab292), 1:2500 α-tubulin (Cell Signaling, #2144). As previously reported, the RhoA antibody detected two bands [27], the lower band is reported to represent active GTP-bound RhoA and has been expressed relative to total RhoA (sum of both bands). Chemiluminescence was used to detect probes and images were quantified using ImageJ 1.44p pixel analysis (US National Institutes of Health).

### Fibrosis

Ventricle samples were fixed in 4% paraformaldehyde and paraffin embedded for histological analysis and cut at 6 μm cross-sections. Masson’s trichrome stain was performed to assess cardiac collagen deposition/interstitial fibrosis (Alfred Pathology, Melbourne, Australia). An Olympus light microscope at 40x magnification was employed to capture images of the LV and the Olympus Image-Pro Plus Version 6.0 was used to enumerate collagen stained blue. To calculate the proportion of fibrosis, the total area of collagen was divided by the total area of the LV and then multiplied by 100%. Data were normalized to a control value of 1 and presented as a fold change.

### Statistics

Statistical analyses were performed using StatView (Version 5.0.1, SAS Institute Inc., Cary, NC, USA). Results are presented as means ± SEM unless otherwise specified. For normally distributed data, the one-way analysis of variance (ANOVA) followed by *Fisher’s* post-hoc test was used to identify differences between groups. Unpaired t-tests were used to compare a single variable between two groups. If data were not normally distributed, the nonparametric Mann-Whitney test was used to identify differences between groups. All relative units are expressed as a fold change with the relevant control group normalized to 1 and a value of P≤0.05 was deemed significant.

## Results

### HSP70 overexpression does not protect against cardiac dysfunction or cardiac conduction abnormalities

**Fig 1:**
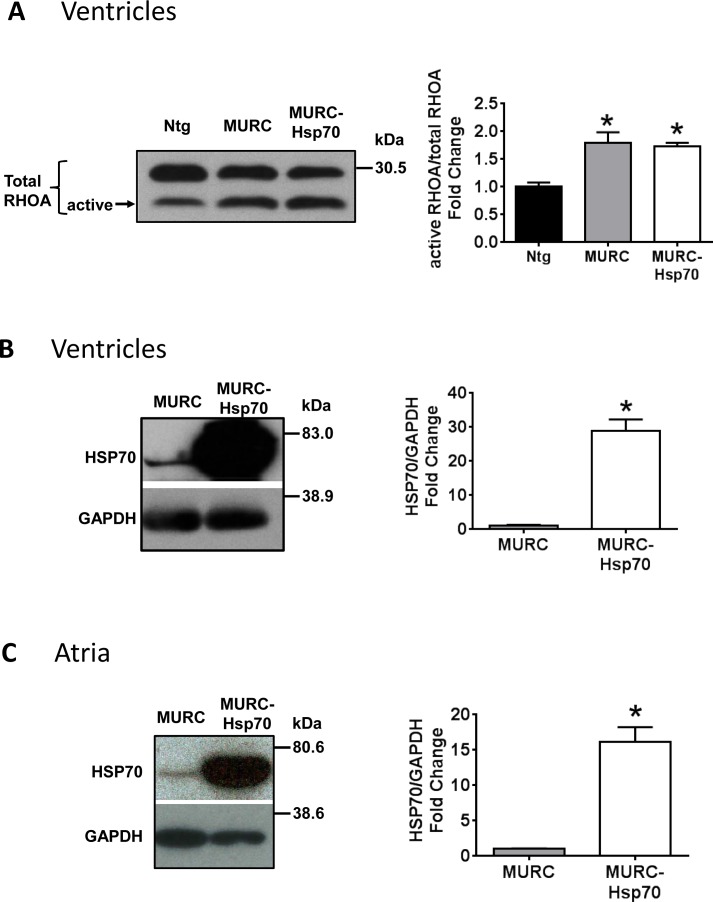
RHOA and HSP70 is upregulated in MURC-Hsp70 Tg mice. **(A)** Representative Western blots and quantification of active RHOA relative to total RHOA (sum of active and inactive RHOA) in ventricles of Ntg, MURC and MURC-Hsp70 Tg mice. N = 3 per group. *P<0.05 vs. Ntg. Representative Western blots and quantification of HSP70 in **(B)** ventricles and **(C)** atria of MURC and MURC-Hsp70 Tg mice. N = 3–5 per group. *P<0.05 vs. MURC.

The main focus of this study was to determine whether increased levels of Hsp70 could mediate protection in a mouse model which develops HF and arrhythmia. Cardiac-specific MURC Tg mice, which develop dilated cardiomyopathy, HF and display atrial arrhythmias [25], were crossed with Hsp70 Tg mice to generate MURC-Hsp70 Tg mice. Transgenic expression of MURC was previously shown to increase RhoA signaling in the heart [25], and this was confirmed in MURC and MURC-Hsp70 Tg mice (Fig 1A). Western blot analysis confirmed that HSP70 expression was substantially increased in both ventricle and atria of MURC-Hsp70 Tg mice compared to MURC Tg mice (Fig 1B & 1C). HSP70 expression also tended to be higher in atria (~2.1 fold, not significant) and ventricle (~8.3 fold, P<0.05) of MURC Tg compared with Ntg, as previously reported in cardiac disease settings [28].

It was previously reported that only ~42% of MURC Tg mice display cardiac arrhythmia up to 5 months of age [25], whereas by 11–12 months, all mice display arrhythmia [22]. Furthermore, there were no significant differences in morphology between Ntg and MURC Tg at 16 weeks of age (heart, atria or lung weights; S1 Fig). Therefore, we studied MURC and MURC-Hsp70 Tg mice at approximately 12 months of age when all MURC mice display ECG abnormalities and morphological changes compared to Ntg. As animals were to be studied after long term housing, and there is an increasing recognition that environmental factors within animal facilities can have on impact on mouse phenotypes [29], it was considered important to assess mice from 3 different cohorts in which there were always MURC and MURC-Hsp70 Tg within the same litter i.e. housed under identical experimental conditions. While these criteria limited the number of mice suitable for the study, we considered this experimental design important for improving the reliability of the reported results. The initial experimental cohort included 11 MURC Tg/MURC-Hsp70 Tg mice. Two MURC-Hsp70 Tg died suddenly 1–2 weeks prior to functional assessment possibly due to a fatal arrhythmia or stroke. On autopsy, both MURC-Hsp70 Tg had very enlarged atria containing thrombi (total atria weight including thrombi: 88 and 51mg; thrombi alone: 41 and 35mg). No MURC Tg died before the study end.

**Fig 2:**
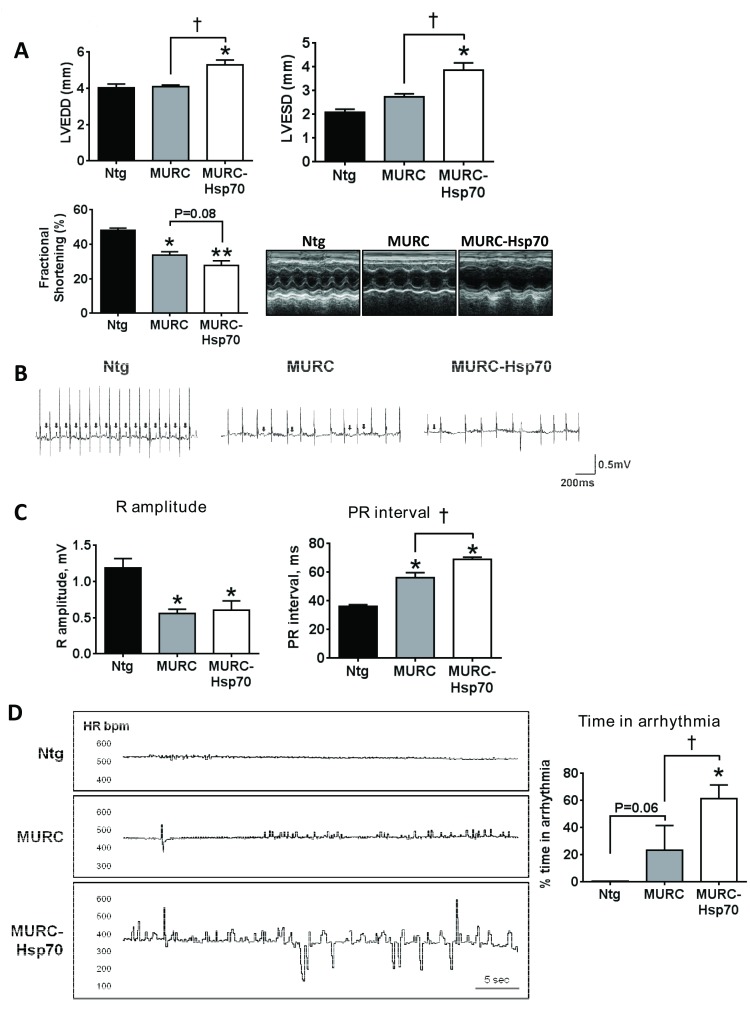
Transgenic overexpression of HSP70 did not attenuate cardiac dysfunction and electrophysiology abnormalities. **(A)** Quantification of LVEDD, LVESD and FS in 12 month old Ntg, MURC and MURC-Hsp70 Tg mice. N = 3–5 per group. *P<0.05 vs. Ntg, **P<0.0005 vs. Ntg, †P<0.05. Lower right: Representative M-mode echocardiograms in 12 month old Ntg, MURC and MURC-Hsp70 Tg mice. **(B)** Representative ECG traces in 12 month old Ntg, MURC and MURC-Hsp70 Tg mice. Arrows highlight clear P-waves. **(C)** Quantification of R amplitude and PR interval in Ntg, MURC and MURC-Hsp70 Tg mice. N = 3–5 per group. *P<0.05 vs. Ntg, †P≤0.05. **(D)** Representative heart rate (HR) variability traces and quantification of time in arrhythmia in 12 month old Ntg, MURC and MURC-Hsp70 Tg mice. N = 3–5 per group. *P<0.05 vs. Ntg (One Way ANOVA with Fisher’s posthoc test), †P<0.05, P = 0.06 (Mann-Whitney nonparametric t-test).

Cardiac dimensions and cardiac function (represented as fractional shortening) were measured in Ntg, MURC and MURC-Hsp70 Tg mice at approximately 12 months of age prior to tissue collection. Fractional shortening was ~30% lower in MURC Tg mice compared with Ntg, and thinning of the ventricular walls was observed (Table 1, see LVPW and IVS). Overexpression of Hsp70 was unable to attenuate cardiac dysfunction and LV remodeling in MURC Tg mice. Interestingly, hearts of MURC-Hsp70 Tg mice were more dilated than MURC Tg mice (LVEDD and LVESD, Table 1 and Fig 2A) and fractional shortening was ~40% lower in MURC-Hsp70 Tg mice compared to Ntg (Fig 2A, Table 1).

**Table 1:** Echocardiography data of Ntg, MURC and MURC-Hsp70 Tg mice at ~12 months of age.

	Ntg	MURC	MURC-Hsp70
**No. of animals**	3	4	5
**BW, g**	45.3±1.2	45.7±1.1	45.3±0.9
**HR, bpm**	532±67	475±25	474±48
**LVPW, mm**	0.98±0.04	0.81±0.04*	0.76±0.02*
**IVS, mm**	1.04±0.04	0.83±0.02**	0.84±0.01**
**LVEDD, mm**	4.05±0.19	4.09±0.10	5.30±0.26* †
**LVESD, mm**	2.08±0.14	2.73±0.14	3.86±0.31* †
**FS, %**	48±1	34±2*	28±3**

BW: body weight; HR: heart rate; LV: left ventricular; LVPW: LV posterior wall thickness; IVS: interventricular septum thickness; LVEDD: LV end-diastolic dimension; LVESD: LV end-systolic dimension; FS: fractional shortening. Data are shown as mean ± SEM. One way ANOVA followed by Fisher’s posthoc test.

*P<0.05 vs. Ntg

**P<0.0005 vs. Ntg

†P<0.05 vs. MURC Tg

Cardiac conduction was examined by direct ECG. ECG traces from MURC Tg mice showed abnormalities including reduced R-amplitude, prolonged PR intervals, and arrhythmia compared with Ntg (Fig 2B–2D, Table 2), as previously described [22]. Overexpression of Hsp70 was unable to attenuate these abnormalities, and Hsp70 had an additional adverse effect on PR interval and time in arrhythmia (Fig 2B–2D, Table 2).

**Table 2:** Electrocardiography data of Ntg, MURC and MURC-Hsp70 Tg mice at ~12 months of age.

	Ntg	MURC	MURC-Hsp70
**No. of animals**	3	4	5
**HR, bpm**	496±17	440±18	419±22
**RR, ms**	121±4	138±6	146±8
**PR, ms**	36±1	56±4*	69±2* †
**QRS, ms**	9±0	10±0	11±1
**R-amplitude, mV**	1.19±0.13	0.56±0.06*	0.60±0.13*
**% time in arrhythmia**	0.33±0.19	23.58±18.02^	61.59±9.94* †

Data are shown as mean ± SEM. One way ANOVA followed by Fisher’s posthoc test. *P<0.05 vs. Ntg

†P≤0.05 vs. MURC

^P = 0.06 vs. Ntg (Mann Whitney nonparametric t-test)

### HSP70 overexpression was not associated with improved cardiac morphology

**Fig 3:**
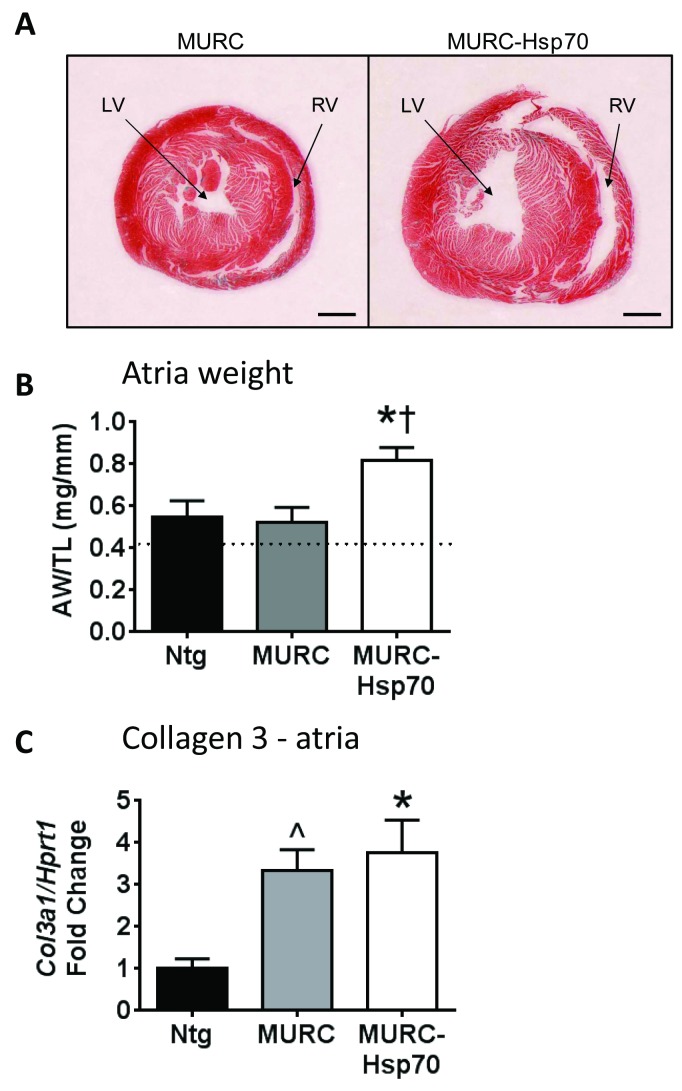
Cardiac morphology in MURC and MURC-Hsp70 Tg mouse models. **(A)** Transverse sections of hearts highlighting dilated chambers in MURC-Hsp70 Tg mice compared to MURC Tg mice. LV = left ventricle, RV = right ventricle. Scale bar = 1 mm. **(B)** Graph of atria weight/tibia length (AW/TL). N = 3–5 per group. *P<0.05 vs. Ntg, †P<0.05 vs. MURC. The dotted line reflects normal AW/TL for Ntg mice at about 3–4 months of age. **(C)** qPCR analysis of Collagen 3 *(Col3a1)* relative to *Hprt1* in atria. N = 3–5 per group. *P≤0.05 vs. Ntg (One way ANOVA with Fisher’s posthoc test), ^P<0.05 vs. Ntg (unpaired t-test).

Total heart weight normalized to body weight or tibia length was not significantly different between groups (Table 3). However, based on echocardiography parameters and at dissection, cardiac dimensions were very distinct; MURC-Hsp70 Tg displayed very dilated chambers (Table 1, Fig 3A). Atrial enlargement is a recognized marker of cardiac pathology but it is also known to increase in size with aging. In comparison to atrial weights of young adult mice, Ntg and MURC Tg mice displayed atrial enlargement at 12 months of age (Fig 3B; dotted line represents a typical ratio for young adult mice). Atrial weight normalized to tibia length was increased further in MURC-Hsp70 Tg compared with both Ntg and MURC Tg (Fig 3B). Consistent with atrial pathology in the MURC Tg mouse models, collagen 3 expression was elevated in atria from MURC and MURC-Hsp70 Tg mice but not Ntg (Fig 3C). Interstitial fibrosis (excessive collagen deposition) in the atria is an important contributor to the AF substrate; interfering with local atrial conduction [30, 31].

**Table 3:** Morphological data for Ntg, MURC and MURC-Hsp70 Tg mice at ~12 months of age.

	Ntg	MURC	MURC-Hsp70
**No. of animals**	3	4	5
**Age, weeks**	53±0.0	53±0.3	53±0.2
**BW, g**	43.7±0.8	43.8±1.3	43.9±0.6
**TL, mm**	17.1±0.1	17.2±0.1	17.2±0.0
**HW, mg**	159.0±5.2	147.4±2.2	178.5±15.4
**AW, mg**	9.3±1.3	9.0±1.3	14.0±1.1* †
**HW/BW, mg/g**	3.64±0.09	3.37±0.08	4.06±0.31
**AW/BW, mg/g**	0.21±0.03	0.21±0.03	0.32±0.02* †
**HW/TL, mg/mm**	9.33±0.35	8.57±0.10	10.40±0.89
**AW/TL, mg/mm**	0.55±0.08	0.53±0.07	0.82±0.06* †

BW: body weight; HW: heart weight; AW: atria weight; TL: tibia length; HW/BW: heart weight/ body weight ratio; AW/BW: atria weight/ body weight ratio; HW/TL: heart weight/ tibia length ratio; AW/TL: atria weight/ tibia length ratio. Data are shown as mean ± SEM. One way ANOVA followed by Fisher’s posthoc test.

*P<0.05 vs. Ntg

†P<0.05 vs. MURC Tg

### HSP70 overexpression did not reduce cardiac fibrosis or improve the cardiac molecular signature

**Fig 4:**
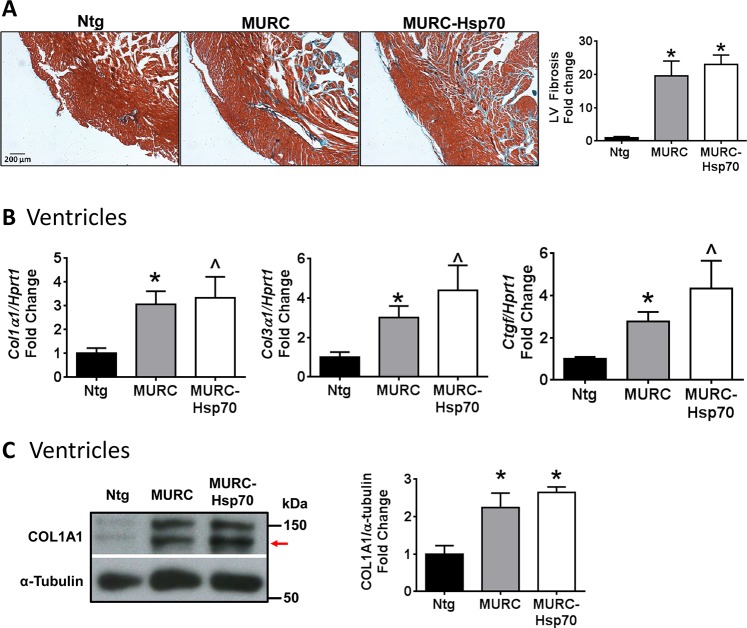
Analysis of cardiac fibrosis and collagen gene expression in MURC and MURC-Hsp70 Tg mouse models. **(A)** Representative LV cross-sections stained with Masson’s trichrome and quantification of LV fibrosis in Ntg, MURC and MURC-Hsp70 Tg mice. Scale = 200 μM. N = 3–5 per group. *P<0.05 vs. Ntg. **(B)** qPCR analysis of Collagen 1 *(Col1α1)*, Collagen 3 *(Col3a1)* and Connective tissue growth factor *(Ctgf)* relative to *Hprt1*. N = 3–5 per group. *P<0.05 vs. Ntg (unpaired t-test), ^P<0.05 vs. Ntg (Mann Whitney nonparametric test). **(C)** Representative Western blots and quantification of collagen 1 (Col1α1) in ventricles of Ntg, MURC and MURC-Hsp70 Tg mice. Arrow indicates band quantified. N = 3–5 per group. *P<0.05 vs. Ntg.

Pathological cardiac remodeling and HF is typically associated with i) changes in the cardiac molecular signature (e.g. increased expression of cardiac fetal genes including atrial natriuretic peptide *(Anp)* and B-type natriuretic peptide *(Bnp)*, as well as decreased expression of genes important for maintaining cardiac function such as sarcoplasmic reticulum Ca^2+^ ATPase *(Serca2a)* and alpha myosin heavy chain (*αMHC*)), and ii) increased deposition of collagen (fibrosis) in the extracellular matrix which makes the heart stiff [32–35]. On histological analysis, hearts (ventricle) from both MURC and MURC-Hsp70 Tg mice displayed significant cardiac fibrosis (Fig 4A) and this was accompanied with increased profibrotic gene expression including collagen 1, collagen 3 and connective tissue growth factor *(Ctgf)* in the ventricle (Fig 4B). Further, collagen 1 protein was increased in the ventricles of MURC and MURC-Hsp70 Tg mice (Fig 4C). Increased expression of Hsp70 was unable to attenuate ventricular fibrosis and collagen expression (Fig 4A–4C).

**Fig 5:**
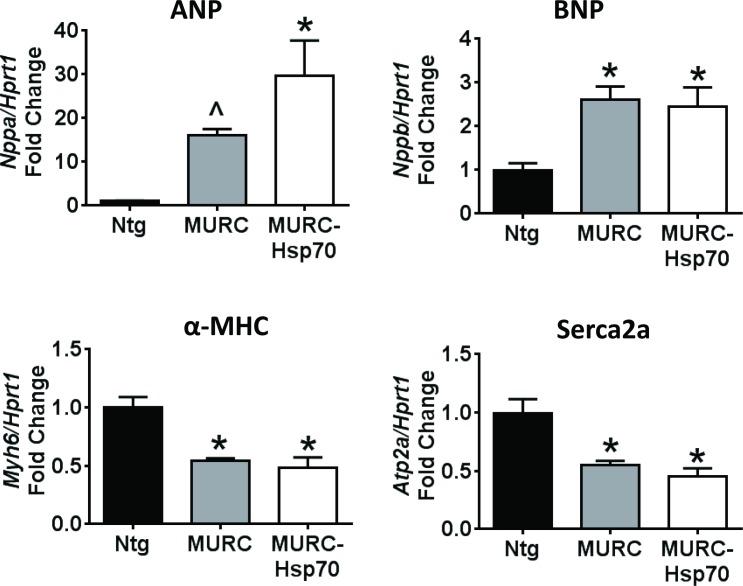
Overexpression of HSP70 does not improve the cardiac gene molecular signature. qPCR analysis of ANP *(Nppa)*, BNP *(Nppb)*, α-MHC *(Myh6)* and Serca2a *(Atp2a)* relative to *Hprt1*. N = 3–5 per group. *P<0.05 vs. Ntg. One way ANOVA with Fisher’s posthoc test. ^P<0.05 vs. Ntg (unpaired t-test).

Cardiac dysfunction observed in the MURC Tg model was associated with significant increases in *Anp/Nppa* and *Bnp/Nppb*, as well as decreased *Serca2a/Atp2a* and αMHC/Myh6 gene expression (Fig 5). Overexpression of Hsp70 was not associated with an improved cardiac molecular signature in MURC-Hsp70 Tg mice (Fig 5).

### Long-term overexpression of Hsp70 alone does not cause cardiac pathology

**Fig 6:**
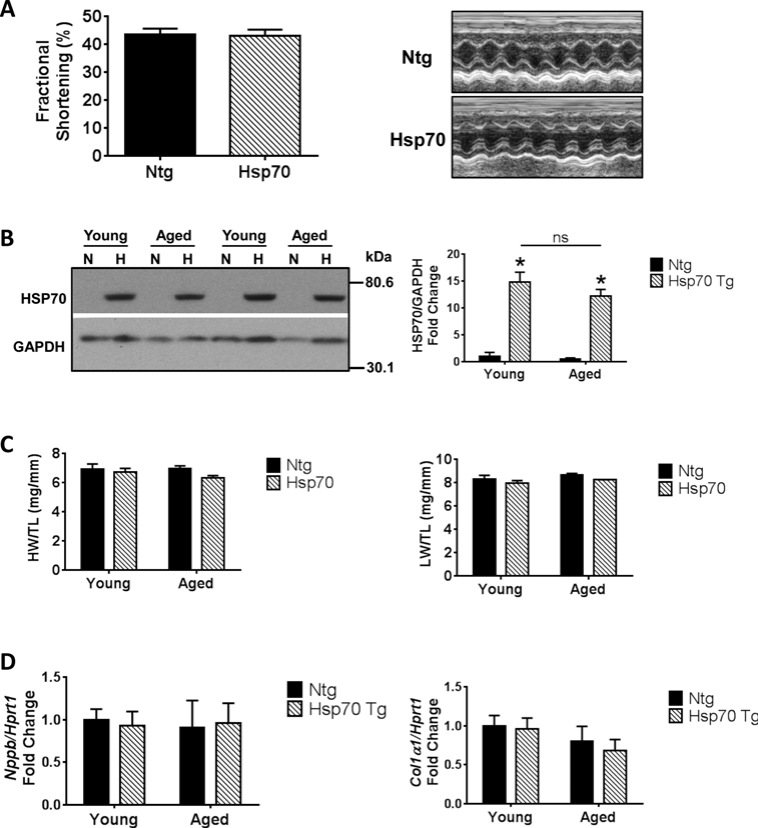
Overexpression of Hsp70 for 12 months does not cause cardiac pathology **(A)** Quantification of FS (left) and representative M-modes (right) in 12 month old Ntg and Hsp70 Tg mice. N = 3 per group. **(B)** Representative Western blots and quantification of HSP70 in ventricles of young and aged Ntg (N) and Hsp70 Tg (H) mice. N = 3–6 per group. *P<0.05 vs. Ntg of same age. ns = not significant. **(C)** Graph of heart weight/tibia length (HW/TL) and lung weight/tibia lenth (LW/TL) of young and aged Ntg and Hsp70 Tg mice. N = 3–6 per group. **(D)** qPCR analysis of BNP *(Nppb)* and collagen 1 *(Col1α1)* in ventricles of young and aged Ntg and Hsp70 Tg mice. N = 3–6 per group.

To assess the possibility of long-term high expression of Hsp70 alone contributing to pathology in the MURC-Hsp70 Tg mice, we characterized a group of Hsp70 Tg mice at 12 months of age. Aged 12 month old Hsp70 Tg mice displayed no evidence of cardiac dysfunction compared to aged Ntg mice (Fig 6A and Table 4). HSP70 protein expression was significantly elevated to a similar degree in both young and aged Hsp70 Tg mice compared to Ntg mice (Fig 6B). Morphological readouts including heart and lung weights (Fig 6C), and molecular makers (BNP and collagen 1) also showed no evidence of cardiac pathology in aged Hsp70 Tg in comparison to aged Ntg or younger animals (8–12 weeks) (Fig 6D).

**Table 4:** Echocardiography data of Ntg and Hsp70 Tg mice at ~12 months of age.

	Ntg	Hsp70
**No. of animals**	3	3
**BW, g**	49.4±0.6	52.4±0.6
**HR, bpm**	538±15	549±52
**LVPW, mm**	0.81±0.01	0.84±0.01
**IVS, mm**	0.88±0.02	0.85±0.01
**LVEDD, mm**	3.89±0.08	3.89±0.06
**LVESD, mm**	2.21±0.12	2.20±0.14
**FS, %**	44±2	43±2

BW: body weight; HR: heart rate; LV: left ventricular; LVPW: LV posterior wall thickness; IVS: interventricular septum thickness; LVEDD: LV end-diastolic dimension; LVESD: LV end-systolic dimension; FS: fractional shortening. Data are shown as mean ± SEM.

## Discussion

While it is generally well accepted that heat shock/hyperthermic treatment or increased expression of Hsp70 via a transgenic or pharmacological approach provides protection in acute cardiac stress settings such as ischemia or induced cardiac arrhythmia [1, 9, 14, 36], it is less established whether an increase in Hsp70 provides benefit in a chronic cardiac disease setting. We recently reported that overexpressing Hsp70 in a mouse model which develops HF and intermittent AF due to expression of Mst1 and dnPI3K provided no protection based on functional, histological and molecular readouts [22]. The goal of the current study was to assess the effect of Hsp70 overexpression in a mouse model that develops HF and AF due to a different mechanism of action i.e. overexpression of MURC. We report here, that overexpression of Hsp70 in a second mouse model which is characterized by cardiac dysfunction, dilatation, arrhythmia and cardiac fibrosis, also provides no benefit.

Approximately 20 years ago, 3 independent laboratories generated transgenic mice with constitutive expression of human or rat inducible Hsp70 under the control of a β-actin promoter (human or chicken) [7, 9, 10]. In each study, hearts were isolated and assessed in a Langendorff perfused apparatus under settings of ischemia (6–30 min) and reperfusion (30–120 min). Transgenic hearts were protected against ischemic injury based on readouts including contractile force, metabolic readouts and infarct size [7, 9, 10]. Our study utilized one of the aforementioned Hsp70 Tg mouse models which was generated by Marber and colleagues (rat Hsp70 with the chicken β-actin promoter) [9]. In this Hsp70 Tg model, Hsp70 is elevated in the heart during development [37]. In contrast, in the MURC Tg model, the expression of MURC occurs immediately after birth (driven using the α-MHC promoter). Despite early and robust expression of Hsp70 in hearts of MURC Tg mice before MURC expression induces pathology, MURC-Hsp70 Tg mice were not protected against cardiac dysfunction, arrhythmia or fibrosis. Notable differences between the earlier mouse studies and our current work include: 1) the model being examined (ischemia-reperfusion vs. HF and AF), 2) the duration of the cardiac insult (acute vs. chronic), and 3) the mode of investigation (ex vivo vs. in vivo).

More recently it was reported that transgenic mice overexpressing Hsp70 were protected against doxorubicin-mediated HF [38]. This group used the transgenic mouse initially described by Pluimer et al. 1995 [10]; also described in Angelidis et al. 1996 [39]. The functional readout used to support this conclusion was protection against a fall in systolic function in Hsp70 Tg mice compared to wildtype/Ntg mice at 6 and 10 weeks post doxorubicin. However, the potential significance of this finding is difficult to ascertain. Systolic function, assessed by measuring fractional shortening on echocardiography, is a heart rate dependent measure, and heart rate was not reported for the different groups of mice. Based on the echocardiographic M-mode images presented within the paper, heart rate was significantly slower in the wildtype/Ntg mouse administered doxorubicin compared with the Hsp70 Tg mouse administered doxorubicin [38]. A faster heart rate in Hsp70 Tg mice could, at least in part, explain the better cardiac function in doxorubicin-treated Hsp70 Tg mice.

The current study, together with our previous work [22], provides no suggestion that increasing Hsp70 in the heart is able to attenuate the pathology that accompanies HF and AF. In fact on some parameters, overexpression of Hsp70 seemed to be associated with worse outcomes (e.g. ventricular dimensions, PR interval, arrhythmia, atrial weight). The explanation for these findings are currently unclear but may be related to the age of the animals studied, and the signaling pathways dysregulated in the stress/disease setting. In the present study, we examined MURC Tg mice at approximately 12 months of age. It is possible that overexpression of Hsp70 may not be effective in the aged heart. It is well recognized that a number of signaling cascades are affected/defective in the aging heart [40] including the induction of Hsp70 in a setting of ischemia [41]. Thus, even though Hsp70 was significantly elevated in the heart of the MURC Tg model, this may be inadequate to provide protection if the activation of other signaling mediators is compromised. To confirm that long-term overexpression of Hsp70 alone was not causing any cardiac pathology, we assessed a cohort of 12 month old Hsp70 Tg mice. Based on cardiac function, morphological and molecular readouts, high chronic expression of Hsp70 does not lead to abnormalities in the heart. Finally, it is noteworthy, that overexpression of Hsp70 also provided no benefit in our previously described HF+ AF model (dnPI3K-Mst1) at 12–13 weeks of age [22].

A limitation of the current study and our previous work [22] is that we were unable to directly assess the effect of Hsp70 overexpression directly on AF. Both the MURC Tg model and dnPI3K-Mst1Tg model display episodic AF, making accurate quantification of AF episodes very challenging. However, given that overexpression of Hsp70 had no positive impact on cardiac morphology, fibrosis or molecular markers, and was associated with premature mortality, greater atrial enlargement, ventricular dilatation and less time in sinus rhythm, there is no reason to think increasing Hsp70 would provide any protection in a setting of HF with or without AF.

Consistent with overexpression of Hsp70 unable to attenuate pathology in a setting of chronic cardiac stress (current study and [22]), we previously reported that Hsp70 Tg mice were not protected in a model of pressure overload with reduced PI3K activity (i.e. aortic-banding of dnPI3K Tg) [42]. Aortic-banded dnPI3K Tg mice displayed a HF phenotype characterized by depressed cardiac function, cardiac fibrosis and pulmonary congestion. These features were not attenuated in aortic-banded dnPI3K-Hsp70 Tg mice [42].

Finally, Hsp70 is not the only Hsp to be linked with human AF or GGA-mediated protection. While a number of studies have provided evidence for an association between Hsp70 expression and protection against AF in humans, other studies have found correlations with other Hsps instead, including Hsp27 and Hsp60 [43–45]. Furthermore, the Hsp inducer GGA has also been shown to induce Hsp27 in addition to Hsp70 [14, 43, 46].

In summary, the current study together with our previous work in another mouse model with HF and AF [22], and a mouse model with HF due to pressure overload and reduced PI3K [42], suggests that an increase in Hsp70 alone is insufficient to provide protection in a setting of chronic cardiac disease. This study has important implications for the future development of Hsp70 based therapies because it illustrates that while this approach may be beneficial in acute cardiac settings, it is not in chronic settings.

## Supporting Information

S1 Fig Morphology of young adult Ntg and MURC mice. Graphs of heart weight/tibia length (HW/TL), atrial weight/tibia length (AW/TL) and lung weight/tibia lenth (LW/TL) of 16 week old Ntg and MURC Tg mice. N = 4 per group.
(TIF)

S1 Table TaqMan® Assays. List of TaqMan® assays used for qPCR analysis.
(PDF)
