# Supplementary figures and images for: Long-Term Overexpression of Hsp70 Does Not Protect against Cardiac Dysfunction and Adverse Remodeling in a MURC Transgenic Mouse Model with Chronic Heart Failure and Atrial Fibrillation
Source: PLoS One. 2015 Dec 14;10(12):e0145173. doi: 10.1371/journal.pone.0145173 (PMC4680216; doi:10.1371/journal.pone.0145173)

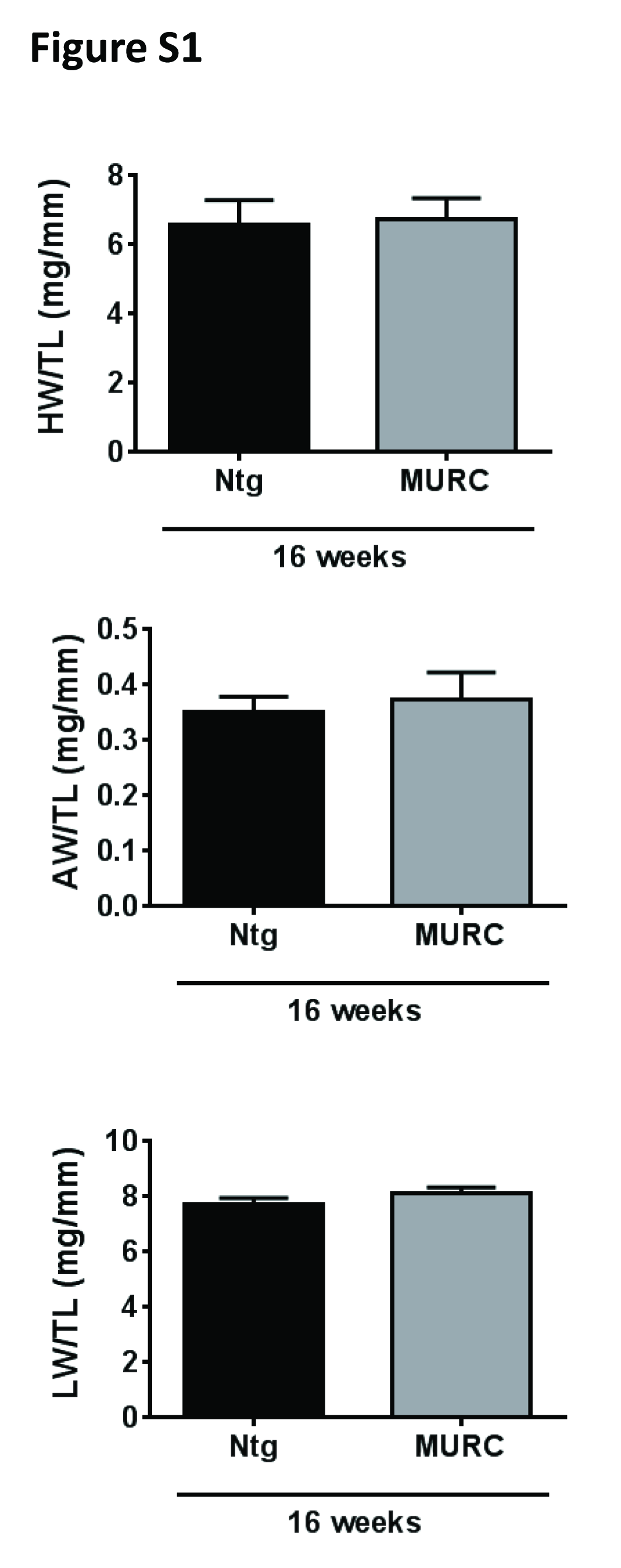

Supplement: S1 Fig — Graphs of heart weight/tibia length (HW/TL), atrial weight/tibia length (AW/TL) and lung weight/tibia lenth (LW/TL) of 16 week old Ntg and MURC Tg mice. N = 4 per group. (TIF) [file pone.0145173.s001.tif]
