# Supplementary material for: Long-Term Overexpression of Hsp70 Does Not Protect against Cardiac Dysfunction and Adverse Remodeling in a MURC Transgenic Mouse Model with Chronic Heart Failure and Atrial Fibrillation
Source: PLoS One. 2015 Dec 14;10(12):e0145173. doi: 10.1371/journal.pone.0145173 (PMC4680216; doi:10.1371/journal.pone.0145173)
Supplement: S1 Table — List of TaqMan® assays used for qPCR analysis. (PDF) [file pone.0145173.s002.pdf]

Table S1: TaqMan® Assays. List of TaqMan® assays used for qPCR analysis.

Gene Symbol	Gene name	TaqMan® Assay ID
Atp2a2	Sarco/endoplasmic reticulum Ca2+-ATPase	Mm01201431_m1
Col1a1	Collagen type 1, alpha 1	Mm0801666
Col3a1	Collagen type 3 alpha 1	Mm01254476
Ctgf	Connective tissue growth factor	Mm01192932_g1
Myh6	α myosin heavy chain	Mm00440359
Nppa	Atrial natriuretic peptide	Mm01255747_g1
Nppb	B type natriuretic peptide	Mm01255770_g1
